# Supplementary figures and images for: The molecular chronology of mammary epithelial cell fate switching
Source: bioRxiv. 2024 Nov 4:2024.10.08.617155. Originally published 2024 Oct 9. Preprint. [Version 2] doi: 10.1101/2024.10.08.617155 (PMC11482796; doi:10.1101/2024.10.08.617155)

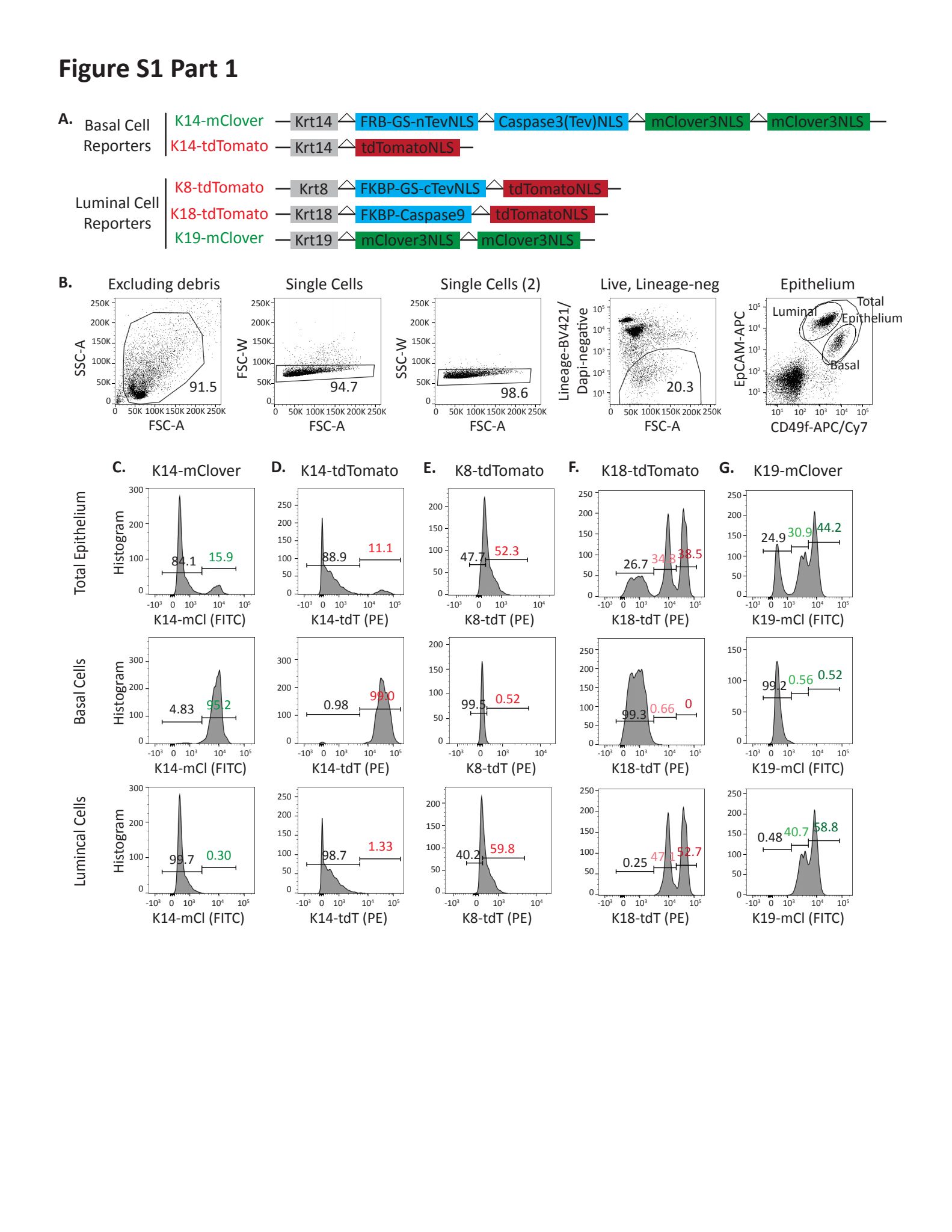

Supplement: Supplement 1 — A. Detailed design of mammary epithelial cell state indicator mice shown in Figure 1A. Basal cell reporters: Keratin14 (Krt14)–mClover (K14-mCl) and Krt14-tdTomato (K14-tdT). Luminal cell reporters: K8-tdT, K18-tdT, and K19-mCl. NLS (nuclear localization sequence) and 3NLS (three consecutive NLS) drive fluorophore nuclear localization for each cell state reporter. Self-cleaving 2A sequences separate each gene, indicated by triangles. When K14-mCl is crossed with K8- tdT or K18-tdT, K14;K8 or K14;K18 double-positive cells can be targeted for caspase-driven cell death by rapamycin- inducible cell killing systems. B. Example gating scheme for identification and isolation of mammary epithelial cells by flow cytometry. Debris, cell doublets, and dead (Dapi+) or lineage+ (CD45+/Ter119+/CD31+) cells are initially excluded. Epithelial cells are then identified as EpCAMHi; CD49fLo (luminal) or EpCAMLo; CD49fHi (basal). C – G. Representative histograms of (C) K14-mCl expression, (D) K14-tdT expression, (E) K8-tdT expression, (F) K18-tdT expression, or (G) K19-mCl expression within total mammary epithelium (top row), basal cells (middle row), or luminal cells (bottom row). [file media-1.jpg]

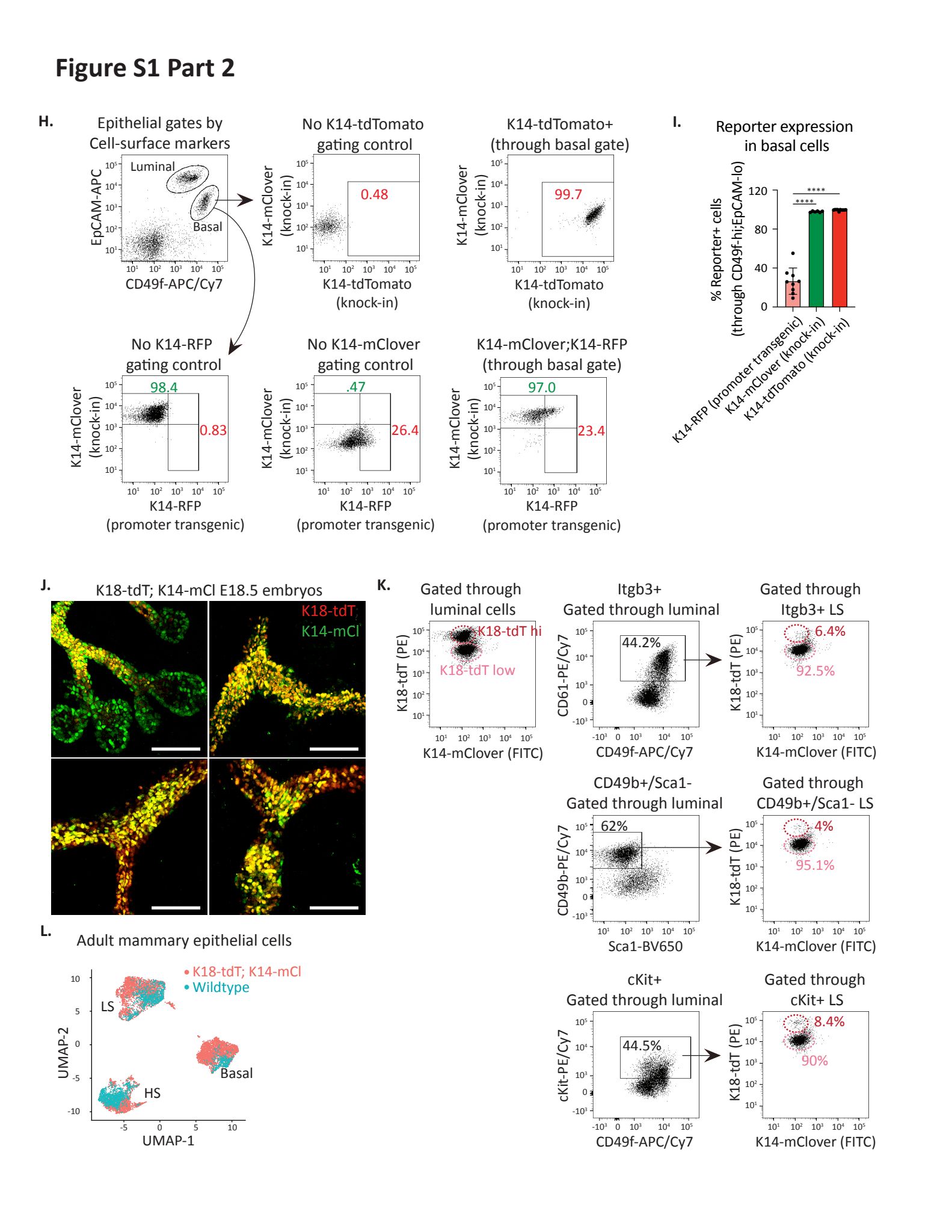

Supplement: Supplement 2 — H – I. Comparison of EpCAMLo; CD49fHi basal cells with knock-in reporter expression (K14-tdT and K14-mCl) or previously published K14-promoter transgenic expression (K14-RFP). Representative flow plots (H) quantified in (I). n = 6–10 per group. J. Representative z-stack images of K18-tdT; K14-mCl dual-reporter endogenous fluorescence in mammary buds from E18.5 female embryos. Yellow cells indicate dual expression of K18-tdT and K14-mCl. Scale bars: 100 μm; z-stacks: 10 μm (top) and 16 μm (bottom). K. Representative dot plots of flow cytometry analysis of K18-tdTHi and K18-tdTLo luminal frequency within alveolar luminal secretory cell populations Itgb3+ (CD61+; top), CD49b+/Sca1− (middle), and cKit+ (bottom). L. UMAP plot of mammary epithelial cells isolated from adult K18-tdT; K14-mCl and wildtype mice analyzed by 3’ scRNA- seq. Each data set is representative of cells pooled from n = 3 mice. Data are represented as mean ± SD. One-way ANOVA with Tukey’s multiple comparison test; **** p ≤ 0.0001 [file media-2.jpg]

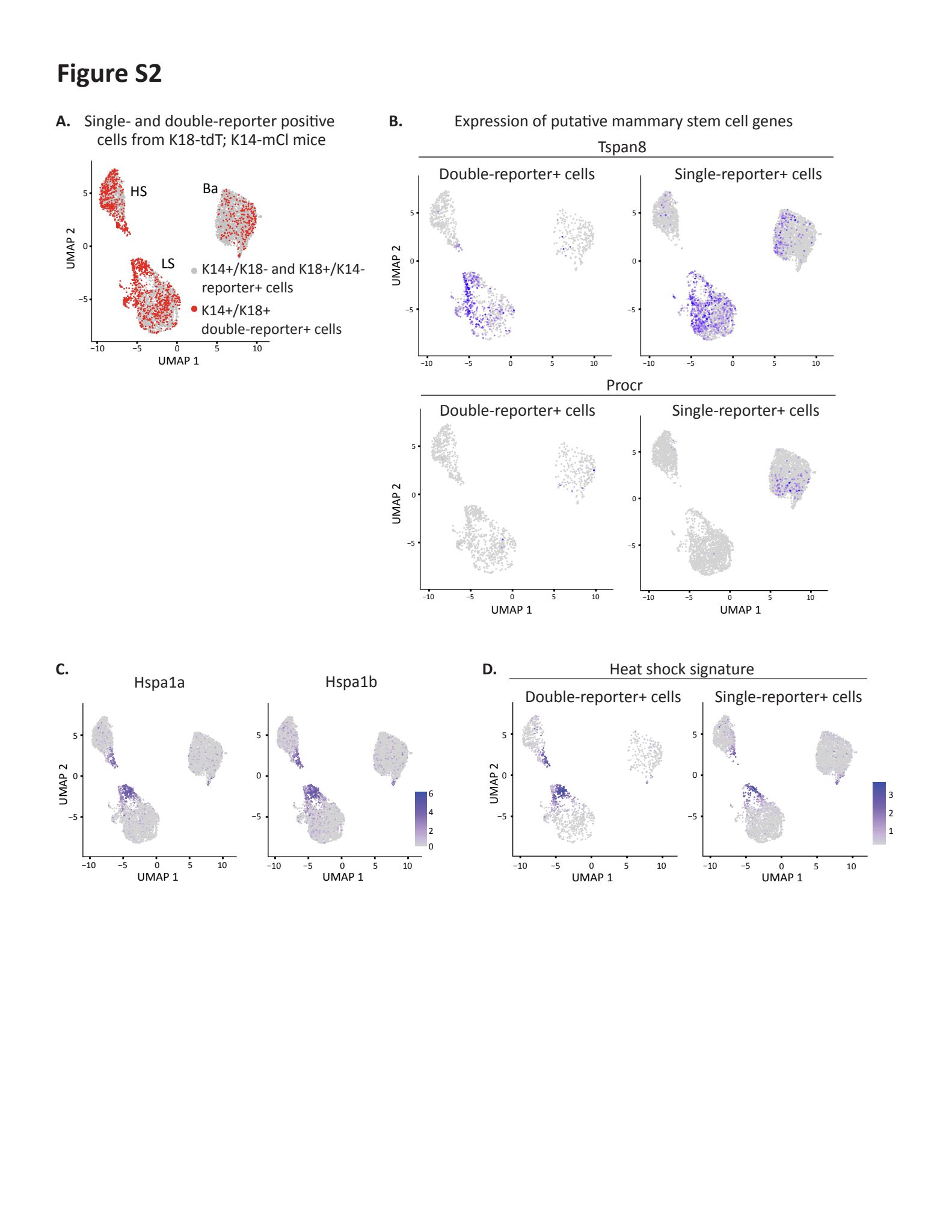

Supplement: Supplement 3 — A. UMAP plot of mammary epithelial cells isolated from adult K18-tdT; K14-mCl analyzed by 3’ scRNA-seq. K18-tdT+/K14-mCl+ double-positive reporter cells (red) are overlayed on single-positive reporter cells (gray). Each data set is representative of cells pooled from n = 3 mice. B. Expression of putative mammary stem cell genes Tspan8 (top) and Procr (bottom) in K18-tdT+/K14-mCl+ double-reporter positive cells (left plots) or K14-mCl+/K18-tdT−, K18-tdT+/K14-mCl− single-reporter positive cells (right plots). C. Expression of heat shock related proteins Hspa1a (left) and Hspa1b (right). D. Heat shock transcriptomic signature overlayed on double-positive reporter cells (left) and single-positive reporter cells (right). [file media-3.jpg]

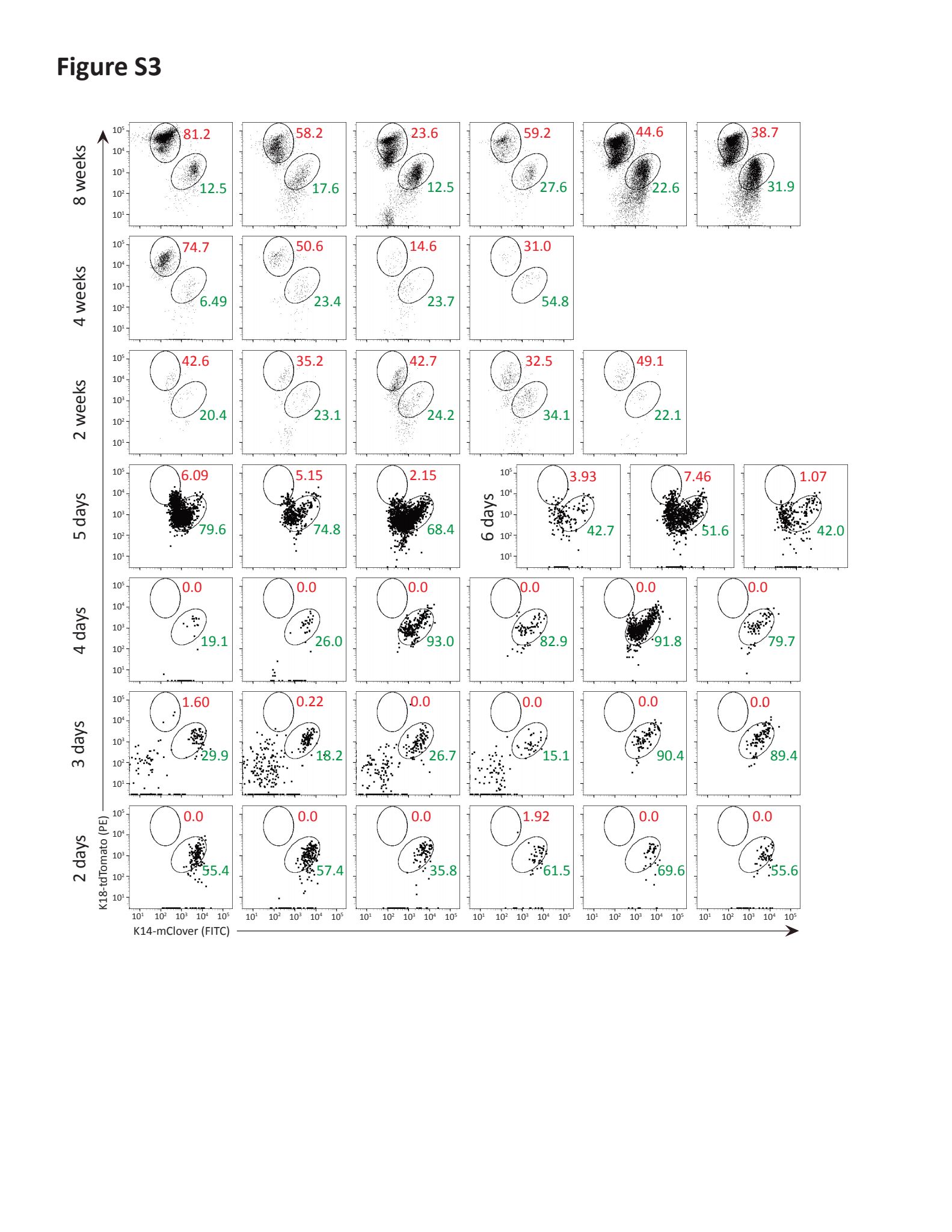

Supplement: Supplement 4 — A. Representative flow plots of reporter expression in transplant recipients at indicated time points. Each plot represents an independent biological replicate. [file media-4.jpg]

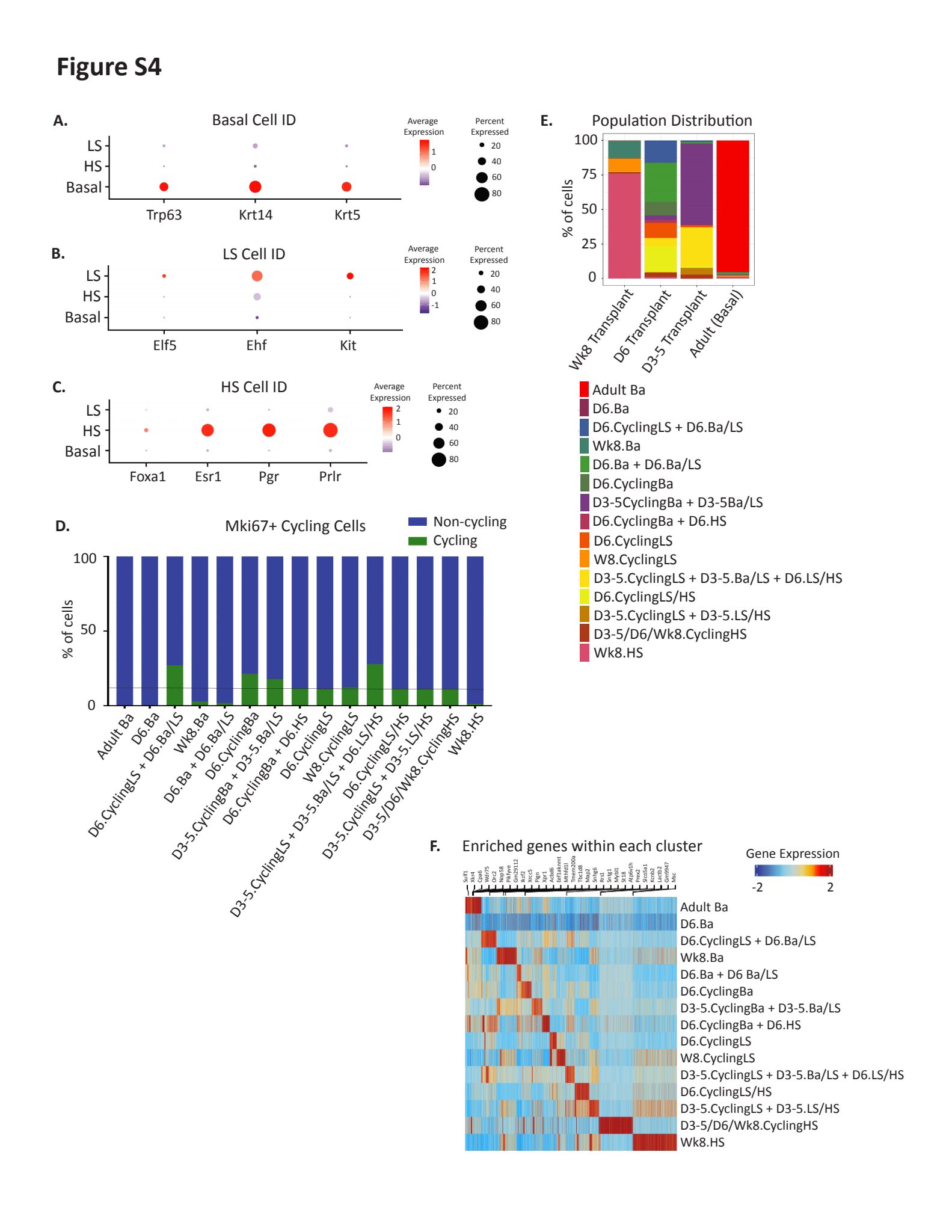

Supplement: Supplement 5 — A-C. Gene expression dot plots of adult basal (A), adult LS (B), and adult HS (C) associated genes. D. Frequency of cells within each cluster expressing Mki67. Cycling cells are defined as Mki67+, and clusters that contain at least 10% Mki67+ are labeled as cycling (indicated by red line). E. Distribution of each cluster comprising Adult Basal, D3–5 transplant, D6 transplant, and Wk8 transplant. F. Gene expression matrix with gene clusters significantly enriched in each cell cluster. [file media-5.jpg]

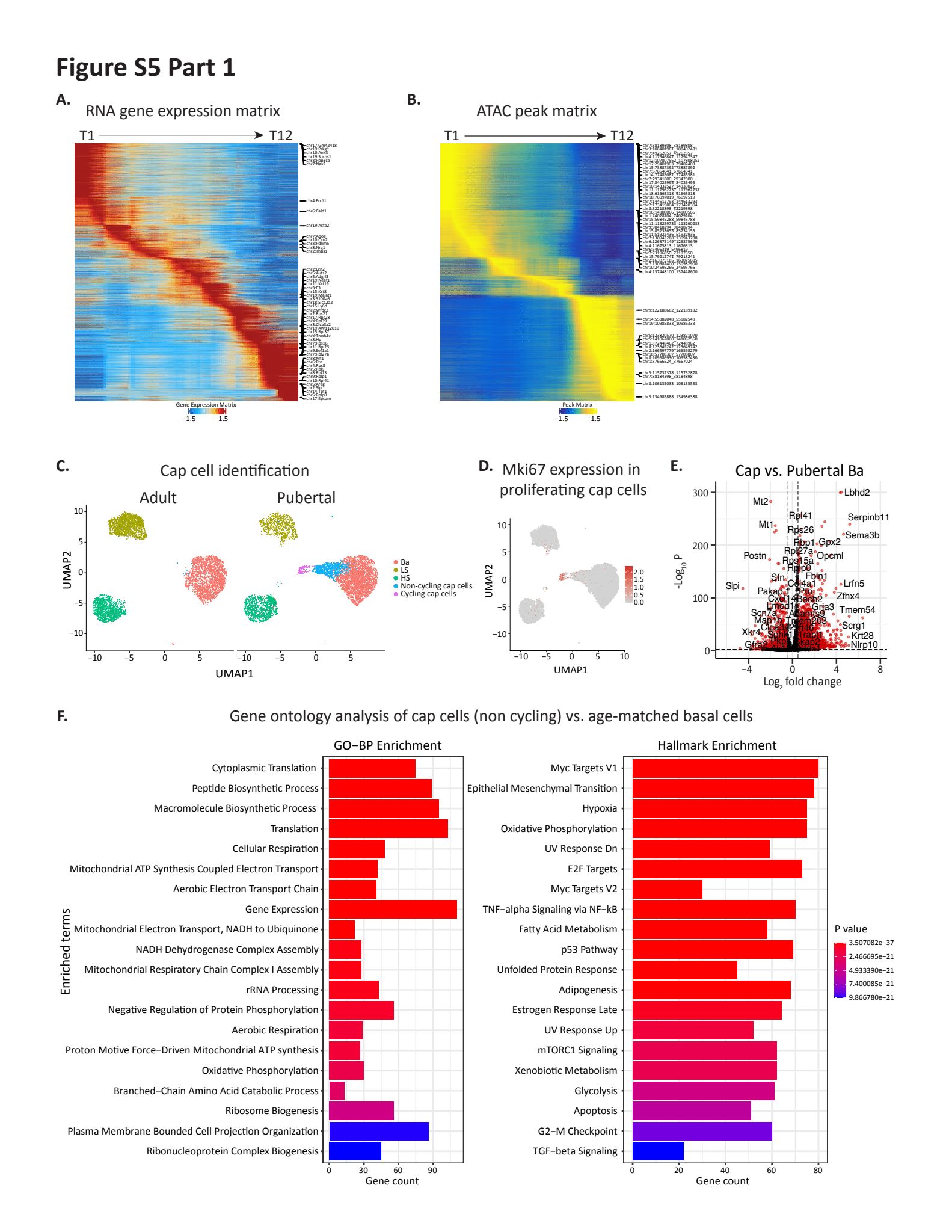

Supplement: Supplement 6 — A-B. RNA gene expression matrix (A) and ATAC peak matrix (B) depicting highly expressed genes or open chromatic regions, respectively, through T1 to T12 transitions identified by Monocle Trajectory analysis. C. UMAP plots showing 3’ scRNA-seq analysis of K14-mCl; K18-tdT mammary epithelial cells isolated from adults (left; 2–4 month old) or pubertal (right; 4–5 weeks old). Non-cycling cap cells (teal) and cycling cap cells (blue) in the pubertal set are observed as a shoulder off of pubertal basal cells (red). Each data set is representative of cells pooled from n = 3 mice. D. Mki67 expression in pubertal K14-mCl; K18-tdT mammary epithelial cells. E. Volcano plot of differentially expressed in non-cycling pubertal cap cells as compared to pubertal basal cells. F. Gene Ontology analysis of non-cycling pubertal cap cell enriched genes vs. pubertal basal cells; Biological Processes (left) and Hallmark gene sets (right). [file media-6.jpg]

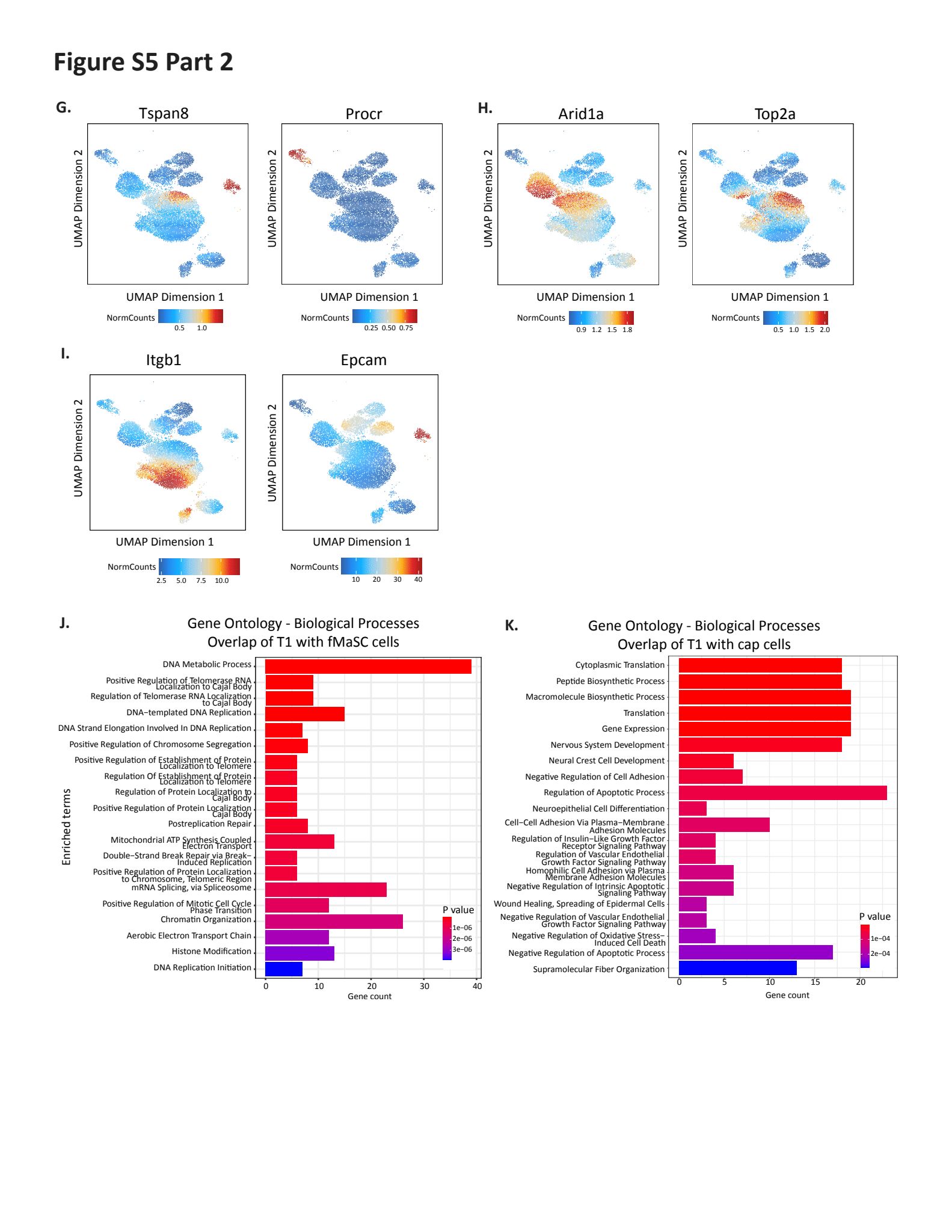

Supplement: Supplement 7 — G. Expression of putative mammary stem cell genes Tspan8 (left) and Procr (right). H. Expression of postnatal chromatin modifiers Arid1a (left) and Top2a (right). I. Expression of Itgb1 (left) and Epcam (right). J-K. Gene ontology analysis highlighting enriched Biological Processes found to co-occur in T1 and fMaSCs (J) or T1 and non-cycling cap cells (K). [file media-7.jpg]

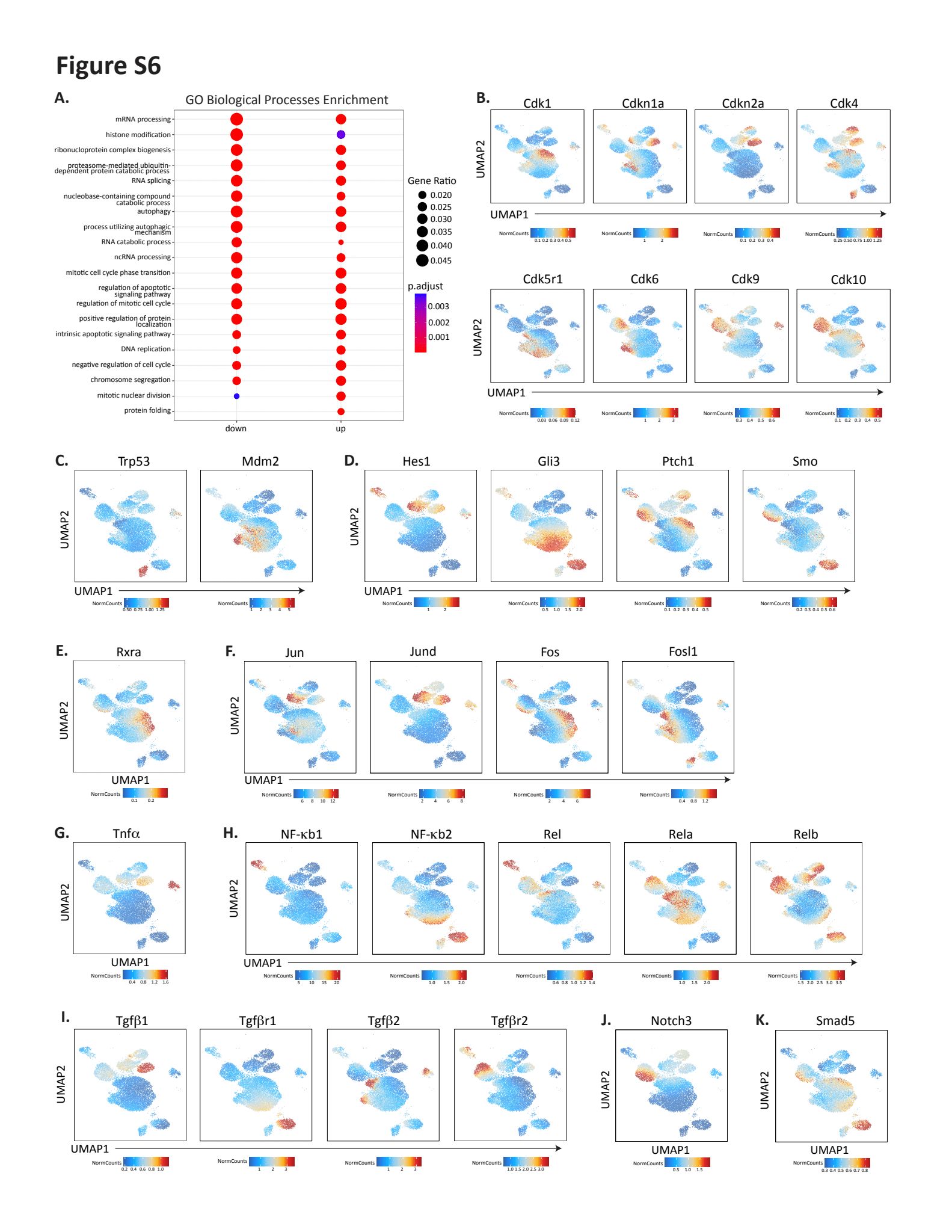

Supplement: Supplement 8 — A. Gene Ontology enrichment analysis of Biological Processes upregulated (right column) or downregulated (left column) in T1 transition as compared to adult basal cells. B-J. UMAP plots showing expression of selected genes involved in key biological pathways, including cyclins (B), p53 (C), Hedgehog (D), RXR (E), AP-1 (F), TNFα (G), NF-κB (H), TGFβ (I), Notch (J), and BMP (K). [file media-8.jpg]
